# The stories behind the statistics

**DOI:** 10.1186/2197-1714-1-2

**Published:** 2014-03-20

**Authors:** Susan P Baker

**Affiliations:** Johns Hopkins University Bloomberg School of Public Health, 624 N. Broadway, Baltimore, MD 21205 USA

**Keywords:** Injury details, Circumstances, Injury prevention

## Abstract

Too often, we fail to illustrate our research findings with descriptions of the circumstances of injury. These details make the subject come alive and provide insight into likely preventive measures. Often the descriptions can be gleaned from accompanying text, relevant newspaper articles, or from visits to the scene of injury. Restricting our findings to cold statistics and numerical data does a disservice to our readers and reduces the likelihood that the research results will have maximum effect.

## Introduction

Analyses of coded data do not give the reader a complete understanding of the cases reported. Additional details, obtained by reading uncoded data or exploring the circumstances of the injuries can provide insight into the nature of the subject and the possibilities for prevention.

Today I want to emphasize the importance of learning the *details* of the cases that underlie the *numbers* that are revealed by our research–looking for stories and ideas, not just statistics, because the details are often more important than the numbers.

When I began my career, I was frustrated when reporters who were interviewing me about my research wanted me to give them details about the circumstances of the cases and the injuries I was studying. At first I thought “If 200 drivers were killed, and if 47% of them were drunk, isn’t *that* interesting enough?”

Finally I realized that in order for the media to find the facts compelling, the research had to begin with knowing the circumstances of those deaths; that’s the kind of information that is rarely available in the coded data that we often rely on for our research. Often the details of the circumstances are necessary if we are going to identify successful preventive measures. Let me give you a few examples:

For the first twelve years after receiving my MPH degree, I worked in the Office of the Chief Medical Examiner of Maryland. One morning I left my office to go down to the basement, as I usually did to see what cases had come in to be autopsied. That day they were about to autopsy a 40-year old man who had died in a crash. If I had been studying coded data for such a case, I would have learned “male, age 40, motor vehicle crash, driver, alcohol .09, Baltimore City.”

But the nurse’s notes that came in with his body gave me important information. After a series of observations, she wrote “1 a.m., patient’s blood pressure has dropped to 70/40; I have called Dr. Blank again.” Dr. Blank (not his real name) was on call at the emergency room of this small rural hospital, and the nurse’s notes conveyed the urgency of the situation– but by the time he arrived an hour later the patient had died. I put down the report with the nurse’s notes and watched as the medical examiner opened the abdomen. A huge amount of blood welled from a ruptured spleen; the patient had died from an injury that might have been survivable with prompt care.

This wasn’t the first such case from a rural emergency room, and it made me wonder whether there was anything unique about these cases. So I assembled a small team of two trauma surgeons, Robert Rutherford and Rudy Gertner, and the assistant chief medical examiner, Werner Spitz, and asked them to review a series of 33 fatal isolated abdominal injuries from car crashes. They determined that half of the injuries were probably survivable with reasonably prompt and appropriate treatment. The paper we wrote described the errors and deficiencies in patient care, and the fact that such cases rarely came to the morgue from the large hospitals where trauma surgeons were available around the clock. We recommended that injured patients be taken to hospitals that were equipped and staffed to handle their injuries in a timely manner (Gertner et al. 1972).

Later, the famous Dr. RA Cowley, who was head of Shock-Trauma at University of Maryland hospital, said this small study gave him the ammunition he needed to get the first statewide trauma system in the country, a system that would take trauma victims to the hospitals that were staffed and equipped to handle them, rather than just taking them to the closest hospital (Franklin and Doelp 1980).

In the course of studying motor vehicle crashes, I sometimes made it a point to visit the actual crash site. When I was doing research on collisions of elderly drivers, I recognized the case of a man I knew, an 85-year-old who had been in pretty good health. In fact, he was driving into town to visit his girlfriend– when he pulled from a side road onto a 4-lane highway and turned left into the second lane of traffic, where he was killed in a head-on crash. I went to look at the site and realized immediately that from where he was emerging onto the highway, it was not obvious that there were two more lanes beyond the wide, unmarked median. It was an easy mistake for an elderly driver to make. We went on to read more police reports and look at other places where older drivers had pulled onto highways, when they were not able to figure out the traffic pattern or to correctly guess the speed of approaching vehicles.

It is not always possible to visit the actual scene of injury, but doing so can be really valuable. When we studied back injuries in municipal workers in Baltimore, our ergonomists asked workers to show them exactly what they were doing when they were injured (Myers et al. 1999). At least one investigator had been very skeptical. He shared a popular opinion that most of the back injuries that were reported as job-related had actually been caused by weekend sports or injuries at home. He changed that opinion when he saw some of the tasks the workers had been required to do, such as loading huge tree trunks or heavy mowing equipment onto trucks by brute force, because the lifting equipment that was scheduled to come was not there by the time they had to finish for the day. Even if some free text *had* been available for these cases, it is not likely that the actual tasks– and the reasons for them– would have been revealed in a coded description of the event.

We have had a chance to do a lot of research on aviation crashes, and the National Transportation Safety Board has generously provided extensive coded data, data that certainly make life easier for an investigator and probably tempt some researchers not to bother to read the long text descriptions of the crashes, because it’s a lot of work and they think they have plenty of information. But without reading those descriptions, we not only would have missed out on some fascinating reading, but would never have recognized some of the problems that contributed to the crashes.

For example, we were studying air taxi pilots who had had two or more crashes in the previous six years, to find out how they differed from pilots who had had only one crash. We found that Alaskan pilots were greatly overrepresented– they accounted for half of the ‘repeaters,’ which is what we called the pilots with two or more crashes. On the basis of that, it would have been easy to surmise that Alaskan pilots were reckless or what some people called “accident-prone.” But it was only when we read the detailed reports that we recognized the unusual aspects of their crashes that set them apart from crashes in the lower 48 states–such as having to land on sandbars or contend with moose on the runway–conditions that made it clear that the repeaters did not have some personal characteristics that got them into trouble but were simply flying in an environment that presented extraordinary challenges (Baker et al. 1995).

In other studies, the vivid text descriptions prompted us to explore hypotheses that we otherwise might have missed. For example, in our study of crashes of sightseeing balloon rides, hard landings were very common. When we read the text descriptions of the hard landings, we found several risk factors for serious and fatal injuries that we would not have known about had we been relying on coded data. In particular, the worst outcomes were associated with balloon roll-overs, bounces, the balloon being dragged by the wind along the ground, and passengers falling from balloons (Baker et al. 2013).

When we studied crashes of sightseeing helicopter flights over Hawaii, one report said it was the pilot’s eighth flight of the day. That sent my colleague, Wren Haaland, searching the internet for news articles about the crash. She found that allegedly this company’s policy was to deny pilots lunch breaks or bathroom breaks and to keep the rotors turning all day. Eight flights a day means you not only have tired pilots, but the pilots may not have time to check out the helicopter between flights or to give adequate briefings to the passengers. According to some of the passengers who survived crashes into the ocean, they had not been briefed on the use of lifejackets or where the life jackets were located—one more example of the kind of information that you don’t find in coded data, and it reinforced our recommendation for more attention to passenger briefings (Haaland et al. 2009).

That last case brings me back to my point about being able to give the media the kind of details that will entice them to write a compelling article. Often a Google search will lead you to a news article about a case that illustrates the point you want to make, a case that can help you to ‘hook’ reporters and your audience–to grab their attention and convince them of the need for the preventive actions you envision.

All of these examples that I have shared with you, and many more, are described in the monograph called “Fifty Favorites,” which presents my recollections of 50 research projects that I especially enjoyed (Baker 2012). I am grateful to all of my colleagues who have contributed so much to my work over the years.

## Conclusion

Descriptions of the circumstances of injury enhance the value of research findings and lead to better understanding of the injury problem and potential solutions.
